# Intelligent Steam Power Plant Boiler Waterwall Tube Leakage Detection via Machine Learning-Based Optimal Sensor Selection

**DOI:** 10.3390/s20216356

**Published:** 2020-11-07

**Authors:** Salman Khalid, Woocheol Lim, Heung Soo Kim, Yeong Tak Oh, Byeng D. Youn, Hee-Soo Kim, Yong-Chae Bae

**Affiliations:** 1 Department of Mechanical, Robotics and Energy Engineering, Dongguk University-Seoul, Seoul 04620, Korea; salmankhalid125@gmail.com (S.K.); akwpfldk@naver.com (W.L.); 2 Department of Mechanical Engineering, Seoul National University, Seoul 08826, Korea; oyt9306@gmail.com (Y.T.O.); bdyoun@snu.ac.kr (B.D.Y.); 3 Korea Electric Power Research Institute, Daejeon 34056, Korea; eldorado@kepco.co.kr (H.-S.K.); ycbaenw@kepco.co.kr (Y.-C.B.)

**Keywords:** waterwall tube, leakage detection, machine learning, optimal sensor selection, steam power plant

## Abstract

Boiler waterwall tube leakage is the most probable cause of failure in steam power plants (SPPs). The development of an intelligent tube leak detection system can increase the efficiency and reliability of modern power plants. The idea of e-maintenance based on multivariate algorithms was recently introduced for intelligent fault detection and diagnosis in SPPs. However, these multivariate algorithms are highly dependent on the number of input process variables (sensors). Therefore, this work proposes a machine learning-based model integrated with an optimal sensor selection scheme to analyze boiler waterwall tube leakage. Finally, a real SPP test case is employed to validate the proposed model’s effectiveness. The results indicate that the proposed model can successfully detect waterwall tube leakage with improved accuracy vs. other comparable models.

## 1. Introduction

Given the growing demand for electricity, the operation of modern power plants must be ever more efficient and reliable [1]. The steam boiler, which converts thermal energy into electricity, is one of the most significant components in a steam power plant (SPP). Approximately 60% of boiler outages are the result of boiler tube failure [2]. Such failure can significantly affect the safe and economical operation of thermal power facilities [3]. Early detection and prediction of boiler tube leakage can assist in scheduling shutdowns and reducing maintenance and labor costs [4].

In a power plant, waterwall tubes extract heat from the furnace and convert water into steam. Waterwall tube leakage is one of the most frequent causes of tube failure in SPPs. Corrosion [5], erosion [6], and fatigue [7] are the general phenomena that cause a decrease in tube wall thickness, which ultimately leads to explosion and leakage in the event of failure. Therefore, in the last decade, numerous attempts have been made to detect boiler waterwall tube leakage using three main approaches, namely the model-based method [8], the knowledge-based method [9], and the statistical analysis method [10]. The model-based method is the traditional method that consists of the process variables’ static and dynamic operations. It provides an effective solution in different fault diagnosis applications. However, in some cases, it cannot provide accurate solutions as it is challenging to develop a valid process mathematical model for some industrial applications. For complex industrial problems and processes with an unknown model, the knowledge-based method and statistical approaches are effective in fault detection applications. These approaches easily handle many process variables (sensors). Employing these multivariate algorithms in power plants for intelligent fault diagnosis can reduce the maintenance time and save significant production losses. The idea of e-maintenance based on multivariate algorithms [11] was recently introduced for intelligent fault detection and diagnosis in SPPs. These algorithms include artificial neural networks (ANNs) and multivariate statistical techniques such as Principal Component Analysis (PCA). These techniques can be employed to eliminate the need for additional tasks and can also assist in efficient monitoring of the status of SPPs [12]. These approaches utilize the operators’ knowledge with rich industrial experiences and use the process control variables (sensors) selected by the experts in power plants for intelligent fault diagnosis. However, sensor redundancy issues can highly affect the performance of these approaches. Therefore, removing the redundant and irrelevant sensors and selecting the optimal sensors which are most sensitive to the fault is necessary. Thus, this study focuses on providing a simple and straightforward methodology to choose the optimal sensors for power plant boiler waterwall tube leakage detection.

E-maintenance mostly relies on acoustic emissions [13] and standard process control variables [14] for leak detection and localization. Zhang et al. [15] developed a three-dimensional algorithm based on a time delay of arrival (TDOA) approach that utilizes acoustic emission technology to detect furnace waterwall tube leakage and localize leaks in a 600 MW power plant. However, this approach requires the installation of expensive devices (acoustic sensors), and it is not effective at detecting small tube leaks. On the other hand, various methods of analyzing process control data are currently being investigated [16,17]. Swiercz et al. [18] proposed a leak detection model based on multiway principal component analysis (MPCA) for boiler riser and downcomer tubes that uses process variables determined by experts. Kornel et al. [19] used ANN to develop models for early tube leak detection that are based on process variables. Jungwon et al. [20] used data from thermocouple sensors mounted on the final superheater (FSH) tube bank for plugging tube detection and identification. As these signals are obtained for the process control system, this method eliminates the need to install expensive devices specifically for the intelligent fault detection system. Furthermore, it has been proven that these process variables can provide sufficient data to detect boiler leaks [21]. Generally, an overwhelming amount of data is collected in a power plant, which makes data processing difficult; it also contains redundant and irrelevant information due to the presence of highly localized, redundant sensors [22]. The studies mentioned above rely solely on experts’ experience for the selection of sensitive input sensors to detect boiler tube leakage. However, this may affect the performance of multivariate algorithms as these algorithms are highly dependent on the number of input sensors. This creates a need to develop an accurate and precise method of determining the optimal sensor arrangement for detection of boiler tube leakage.

In the literature, artificial intelligence-based fault detection techniques are generally merged with feature extraction techniques such as PCA to exclude redundant information [23,24]. However, these techniques are not useful in identifying the root cause of a failure. On the other hand, optimal sensor selection via feature selection techniques can be helpful in identifying the most sensitive input sensors by eliminating redundant sensors and reducing dimensionality [25]. Different feature selection techniques, such as optimization-based feature selection [26], regression-based feature selection [27], and classification-based feature selection, have been attempted [28]. These techniques are computationally expensive for data with a large number of features. To implement feature selection approaches in an SPP, it is necessary to weigh the computational cost and the complexity of the algorithm. The correlation method is a well-known feature selection technique that uses a correlation function to estimate the relationship between pairwise inputs and remove redundant features [29]. Therefore, optimal sensor selection via correlation analysis can be considered the most feasible approach in an SPP due to its ease of implementation and reduced complexity and computational cost.

In this paper, we propose a machine learning-based integrated optimal sensor selection technique for waterwall tube leakage detection in an SPP. The study consists of two main parts. In the first part, optimal sensor selection is performed via correlation analysis to select the most sensitive sensors necessary to detect waterwall tube leakage. In the second part, different supervised machine learning algorithms are utilized for boiler waterwall tube leakage detection. In the end, a real power plant boiler waterwall tube leak scenario is used to validate the proposed model’s effectiveness.

## 2. Significance of the Boiler Waterwall Tube in an SPP

**Figure 1:**
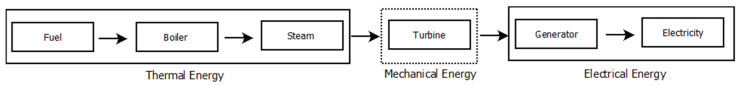
Schematic illustration of energy conversion in a thermal power plant.

In this section, first, a brief introduction to SPPs is given, and then the significance of the boiler waterwall tube in an SPP, including waterwall failure analysis and important monitoring parameters, is discussed in detail. Figure 1 demonstrates the fundamental steps in the conversion of fossil fuels to electricity. The boiler converts fuel energy to heat energy, thus transforming water into steam. In the second stage, the turbine utilizes high-pressure steam to produce electricity [30].

### 2.1. Equipment in a Coal-Fired Power Plant

Modern SPPs are the product of extensive development efforts that have taken place over many years; they rely on state-of-the-art technologies, research work, and experience. However, the essential elements have remained more or less the same through the years, though they have become more efficient as a result of increasingly sophisticated techniques and scientific advancements. The equipment includes five essential elements, i.e., a boiler, turbine, condenser, generator, and monitoring alarm [31].

1. Boiler: A boiler is the primary piece of equipment in an SPP. It transfers energy to water until it becomes a heated steam, which is then utilized to run the steam turbine. The boiler consists of three main subsystems, i.e., a feedwater system, steam system, and fuel/air draft system. Each subsystem comprises numerous additional components that make them suitable for application in advanced power plants.
2. Turbine: The turbine uses high-temperature, pressurized steam to transform heat energy into mechanical energy in order to run the electric generator. The associated subsystems are the turbine gear/barring gear, gland sealing system, and turbine oil system.
3. Condenser: High-temperature steam travels to the condenser from the turbine exhaust outlet. The condenser condenses the steam via heat transfer with cooling water from another source. It includes the steam ejectors, cooling water system, condensate pumps, and heat exchangers as associated subsystems.
4. Electrical generator: The function of an electrical generator is to convert mechanical energy into electrical energy. It includes an exciter and transformer as subsystems.
5. Monitoring alarm system: The alarm system is used to check the health status of the equipment mentioned above. It rings alarms in case of any abnormality.

**Figure 2:**
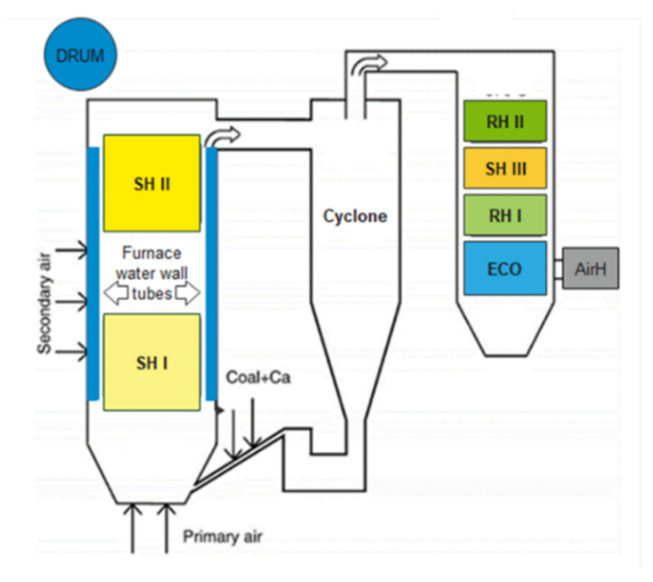
Schematic of a Circulating Fluidized Bed (CFB) boiler in steam power plant.

The current study concerns a circulating fluidized bed (CFB) boiler. CFB boilers have gained popularity due to their various benefits, such as improved combustion efficiency, stable operation, and lower NOx emissions [32]. A CFB boiler incorporates a combustor, solid separator, and second pass flue gas ducting. A solid cyclone separator connected to the outlet of the combustion chamber collects most of the solids leaving the chamber and delivers the remainder back to the combustor. A convective pass includes superheaters, reheaters, an economizer, and air heaters. Water travels around the waterwall tubes to generate steam. The steam then enters the stage I and stage II superheater (SHI and SHII), where it overheats. The primary and final reheater (RHI and RHII) collect steam from the high-pressure turbine in the second pass. Finally, the second-pass flue gas ducting comprises an economizer and air heater (AirH). A schematic diagram of the CFB boiler in steam power plant is shown in Figure 2. 

### 2.2. Waterwall Tube Failure Analysis 

The waterwall tube is one of the most important components of a boiler. Leakage in the waterwall tube is a serious problem, and various studies have been conducted to examine waterwall tube failure. Nurbanasri et al. [33] carried out a microstructural analysis of waterwall tubes and observed that cracks were initiated due to defects that occurred during the welding process. Ahmad et al. [34] investigated the failure mechanisms in rear waterwall tubes. They showed that wall thinning happens due to fly ash erosion and an increase in temperature, which prompts the thermally activated process of creep problem. Moreover, Liu et al. [6] found that wall thinning in the fire-facing side of a waterwall tube occurs due to oxidation of the tube, which causes the tube pressure to surpass the bearing limit of thin tubes.

Condition-based monitoring utilizing a data-driven approach is one possible and efficient solution for fault diagnosis and classification [35]. Data-driven maintenance of the condition of an object can be divided into two main steps: (1) acquisition of data on the relevant status of the object, and (2) data preprocessing and classification of the preprocessed data. Thus, tube leakage detection can be defined as a classification problem. Acoustic emission (AE) is one of the most popular techniques used in boiler tube leak detection [36]. However, AE-based leak detection systems are not effective as they cannot detect small tube leaks [37]. Furthermore, a change in the power plant’s operating conditions generates a significant degree of variation in the characteristics of the signal, and the noise due to the geometry of the furnace, which creates echoes, also affects the outcomes of signal processing, thus complicating the decision process. An alternative technique for data-driven maintenance is to identify the patterns associated with healthy and faulty conditions directly from historical process control data [29]. Such an approach does not require complicated models of large-scale plant operation and eliminates the need to install expensive devices.

In an SPP boiler, it is difficult to pinpoint the exact moment at which a leak occurs, and a significant amount of time elapses between the formation of a small hole and the moment at which the leak is large enough to cause identifiable failure. Moreover, the length of the tube is several dozen meters, and a leak may develop in a random location. Such fault developments impact the process variables in numerous ways. Therefore, a tube leak detection algorithm must employ estimations of the different process variables and associate them with the typical fault patterns.

## 3. Proposed Methodology

**Figure 3:**
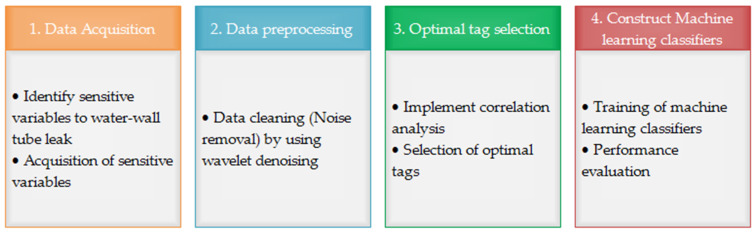
Overview of the proposed machine learning-based optimal sensor selection model.

This section introduces the complete methodology used to implement our machine learning-based optimal sensor selection model. The proposed model is divided into four main stages, as shown in Figure 3. In the first stage, the key monitoring variables that are sensitive to waterwall tube leakage are identified. The second stage is the data preprocessing phase. In the data preprocessing phase, the data are preprocessed to enable optimal sensor selection and construction of machine learning classifiers. This step includes noise removal and data normalization. In the third step, optimal sensor selection based on correlation analysis is utilized to reduce the number of sensor variables for the input data. In the final step, the primary task is to construct the machine learning classifiers and validate the performance of the optimal sensor selection analysis. The following subsections present a more comprehensive analysis of the proposed model.

### 3.1. Data Preprocessing

Real power plant data are generally noisy and inconsistent; data preprocessing is required to overcome these obstacles [38]. Upon analyzing the acquired real plant data, it was observed that the plant data contain noise due to the measuring instruments (sensors) that need to work in harsh environments, e.g., vibration, high temperature, and corrosion [39]. The sensors are more vulnerable to strong electromagnetic interference and the influence of temperature and humidity. During the data transmission process, the signal data are inevitably mixed with the channel noise and the propagation error. All the factors mentioned above result in noise in the monitoring data. The resulting noisy data can affect data analysis and the performance of the machine learning algorithms. Therefore, it was necessary to analyze and preprocess the data obtained from the power plant. 

Recently, wavelets have emerged as a powerful tool for noise removal in fault diagnosis applications [40]. Wavelets can be used to identify important features during noise removal, as different features are localized at different scales [41]. Traditional techniques, such as Fourier transform analysis [42] and power spectral density analysis [43], are more sensitive to impulsive oscillations and cannot be used to obtain hidden frequencies in the data. Wavelet analysis helps to overcome these drawbacks by simultaneously monitoring both the time and frequency domains. Wavelet-based denoising is chosen and preferred over filter-type denoising for multiple reasons. The first and foremost reason is that one needs to know the signal’s frequency content and noise frequency content for filter-type denoising. However, in this study, we could not clearly separate the two, which hindered the filter-type denoising application. Wavelet analysis can analyze the signal in both the time and frequency domain, giving it an advantage over the typical techniques that only focus on one aspect, either time domain or frequency domain. This study’s sensor signals consist of long non-stationary events; the potential noise sources were unknown. Using wavelet is recommended in such scenarios, as wavelet-based denoising is best suited for non-stationary signal analysis. The main advantage of a wavelet basis is that it can perfectly reconstruct functions with linear and higher-order polynomial shapes despite having an irregular shape of the signal. The denoising was performed using the Daubechies wavelet, the most used set of discrete wavelet transforms (DWT). The wavelet coefficients were denoised by using the wavelet thresholding method. The thresholding method is simple and has a good effect on the aspect of removing noise. Soft thresholding performs better when the detailed wavelet coefficient contains both signal and noise [44]. In this study, the wavelet analyzer toolbox in MATLAB [45] was used for denoising by selecting soft thresholding for Daubechies wavelet with five decomposition levels. The wavelet denoising method consists of three steps. In the first step, signals are decomposed using wavelet transform in both the time and frequency domains. Wavelet transform of the continuous signal *x*(*t*) is defined in Equation (1). In the second step, an appropriate threshold limit is selected and a threshold method that optimizes the noise removal process is defined. In the final step, the denoised signal is obtained by taking the inverse wavelet transform of the wavelet coefficients.

(1) $$\mathrm{WT}\left(a,b\right)=\overset{\infty }{\underset{-\infty }{\int }}x\left(t\right)\bar{\psi }\left(\frac{t-b}{a}\right)dt$$

where ψ(*t*) is the analyzing wavelet, a is the scale parameter, and b is the position parameter.

### 3.2. Optimal Sensor Selection 

**Figure 4:**
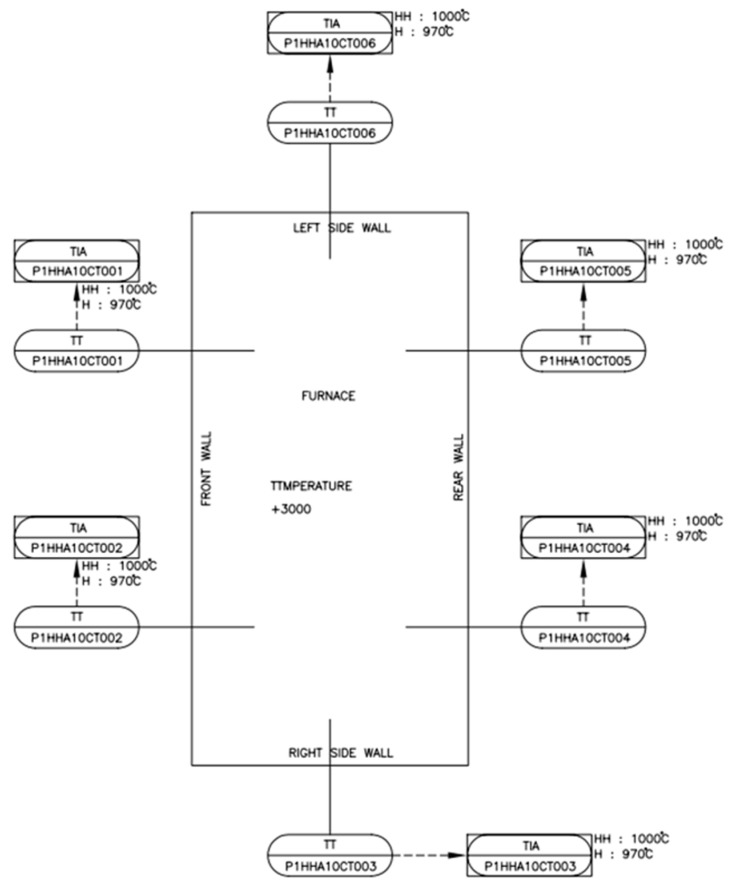
Piping and instrumentation diagram (P&ID) of the furnace section of the boiler.

In a power plant, operators and field technicians use a piping and instrumentation diagram (P&ID) to keep track of all the equipment and the sensors that regulate the process flow. A unique tag number or label is assigned to each sensor for individual identification. For example, in Figure 4, the P&ID diagram of the furnace section shows the six thermocouple sensors with their unique tag numbers that are used to measure the furnace wall temperature at separate locations. These localized sensors may contain irrelevant and redundant information that may influence the performance of a multivariate algorithm. Therefore, it was necessary to decrease the number of process variables (sensors) and pick the optimal number of sensors necessary to detect waterwall tube leaks.

Generally, the data collected in the power plant are too large to handle, and to overcome this issue, artificial intelligence (AI)-based fault detection techniques are commonly combined with feature extraction techniques such as PCA to remove redundant information. Statistically, PCA can reduce the sensor data’s dimensionality, but it is not possible to pinpoint the redundant sensor by using dimensionality reduction techniques. To physically specify the redundant sensor, optimal sensor selection via a feature selection technique such as correlation analysis is the most suitable solution. It allows for identifying the redundant and irrelevant sensors by showing its correlation with other sensors.

In this study, first, the Pearson correlation coefficient [46] (Pearson’s *r* value) is computed between the input sensor variables. Only the highly correlated sensors are retained. The Pearson coefficient values range from −1 to 1, where 1 represents the strongest possible positive correlation, 0 shows that there is no linear correlation, and −1 is the strongest possible negative correlation between two variables. This method evaluates the strength of the relationship between two sensor signals, as shown in Equation (2).

(2) $$r=\frac{s\left(\sum ab\right)-\left(a\right)\left(\sum b\right)}{\sqrt{\sqrt{\left[\left[s\sum b^{2}-{\left(\sum b\right)}^{2}\right]\right]\left[s\sum a^{2}-{\left(\sum a\right)}^{2}\right]}}}$$

where s is the sample size, *r* is the Pearson correlation coefficient, and a and b are the two sensor signals.

### 3.3. Machine Learning Algorithms

**Figure 5:**
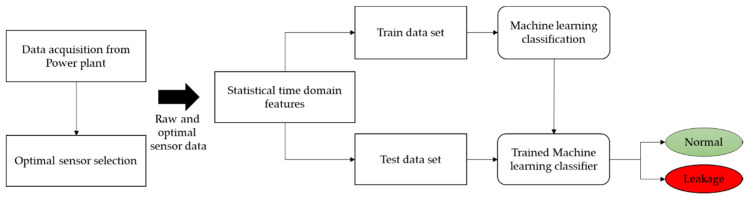
Schematic of the machine learning process used to predict the boiler health state.

Recently, machine learning in intelligent fault diagnosis applications has become an area of intense focus [47]. Machine learning uses examples and the knowledge gained from experience to optimize a task. There are three main types of machine learning techniques: supervised machine learning, unsupervised machine learning, and reinforcement machine learning. Of these, supervised learning is the most popular technique for classification and regression problems. The results of supervised learning are reliable and accurate owing to the use of labeled and well-characterized input data [48]. In this study, four well-known supervised machine learning classifiers (support vector machines (SVMs), k-nearest neighbors (k-NNs), naïve Bayes algorithm (NB), and linear discriminant analysis (LDA)) are employed and compared in terms of their performance. The overall schematic of the machine learning process is shown in Figure 5. The methodology involves employing and evaluating two cases (raw and optimal sensors) in machine learning classifiers. First, the training and testing datasets are created from the individual time domain sensor data. Eighty percent of the sensor data are used for training purposes, and the remaining twenty percent of the data are used as independent testing. Statistical time domain features are extracted and used in machine learning algorithms. The classifiers’ training is carried out by using a 10-fold cross-validation strategy on the training data to avoid overfitting. The independent test dataset is used for the validation of classifier’s performance. The raw and optimal feature dataset is processed, and the performance of the employed supervised learning classifiers are compared. The details of the machine learning classifiers are given as follows:

#### 3.3.1. SVM Classifier

SVM works by establishing a hyperplane (decision boundary) between two classes and attempting to orientate the boundary in a manner such that the gap between the two classes is maximized [49]. The main benefits of SVM include the use of kernels that can solve any complex problem, the smaller risk of overfitting, and the absence of local minima. Because of these advantages, SVM is quite popular in fault detection and isolation problems [50].

Given that n is the total number of experiments in the training dataset, $S={\left\{x_{i},y_{i}\right\}}_{i=1}^{n}$, where $x_{i}\in R^{n}$, $y_{i}\in R$, $y_{i}$ denotes the target value corresponding to $x_{i}$. An SVM attempts to develop a function *f*(*x*) based on the relationship between $\left(x_{i},y_{i}\right)$ that is as smooth as possible by minimizing the error between the target and output values. In the case of linear non-separable data, SVM uses kernel functions, and these kernel functions have a significant influence on the performance of the model. The most commonly used kernel functions are as follows [51]:

1. Linear kernel
  (3) k(x,y)=X.Y
2. Polynomial kernel
  (4) $$k\left(x,y\right)={\left(x.y+1\right)}^{p},\;p=1,2,\;3\ldots ,\;n$$
3. Radial basis function
  (5) $$k\left(x,y\right)=\exp\left\{-\frac{{\left|x-y\right|}^{2}}{2\sigma^{2}}\right\}$$
4. Hyperbolic tangential kernel
  (6) k(x,y)=tanh{kx.y+θ}
  where k>0 and θ<0.

In this paper, RBF is chosen as the kernel function of the SVM due to its superior characteristics, such as stronger robustness, infinite smoothness, and ease of calibration. 

#### 3.3.2. k-NN Classifier

A k-NN classifies its target by measuring the distance between the target and the nearest feature space. Euclidean distance $d_{E}$ is generally used to measure the distance between two points, x and y, with Equation (7).

(7) $$d_{E}=\sqrt{\sum_{i=1}^{n}{\left(x_{i}-y_{i}\right)}^{2}}$$

k-NNs are being used in many applications, such as image processing, pattern recognition, and fault classification. We chose to use k-NN in this work because of its inherent advantages, such as ease of implementation, robustness, and ability to tune the network using few parameters. 

#### 3.3.3. NB Classifier

An NB algorithm is a simple and powerful probabilistic machine learning algorithm for classification based on Bayes’ theorem [52]. An NB algorithm is easy to implement and primarily used for large datasets. The algorithm works on the assumption of conditional independence, i.e., the presence of a feature (*x*) in a class (*c*) is irrelevant and unbiased towards any other features. The conditional independence assumption is shown in Equation (8) [53].

(8) $$P\left(c|x\right)=\frac{P\left(x|c\right)P\left(c\right)}{P\left(x\right)}$$

where P(c) is the prior probability of a given class regardless of the predictor, P(x) is the probability of the predictor regardless of the given class, and P(c|x) is the probability of the predictor given the data, also known as the posterior probability. NB classifiers have been used in many real-world applications. In this study, we have opted to use a naïve Bayes classifier because of its substantial advantages in terms of ease, learning and classification speed, and storage space.

#### 3.3.4. LDA Classifier

LDA is a commonly used multivariate classification method that aims to find a linear combination of features for class separation [54]. It attempts to project higher-dimensional data onto lower-dimensional space to deliver maximum class separability and avoid overfitting and computational cost. Logistic regression [55] and LDA [56] are widely used in pattern recognition in conventional statistical learning techniques. Therefore, LDA is adopted in this study because it requires no parameter tuning and because the extracted features are easier to understand under linear assumptions.

## 4. Real-World Power Plant Scenario—Computational Results

In this section, a real-world case of boiler waterwall tube leakage is employed to verify the effectiveness of the proposed model. 

### 4.1. Acquisition of Leak-Sensitive Sensor Data and Data Preprocessing

This study utilizes the data from 38 sensitive sensors in a SPP: these sensors provide data on the inlet and outlet header temperatures; the tube metal temperature, which is collected from thermocouples mounted on the superheaters (SHI, SHII, and SHIII) and reheaters (RHI and RHII); and the active power of the corresponding generator. Table 1 summarizes the most sensitive sensors in the SPP; in the table, “ID” represents the number assigned to each sensor and a notation is assigned to each sensor to facilitate the optimal sensor selection process.

**Figure 6:**
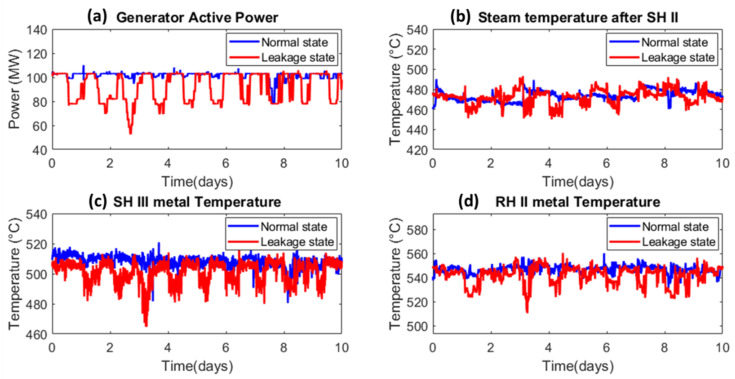
Effect of tube leakage on leak-sensitive variables (**a**) Generator active Power (**b**) Steam temperature after SH II (**c**) SH III metal temperature (**d**) RH II metal temperature.

Figure 6 presents the trends in active power when the plant is in healthy (fully functional) and leakage states, SHIII metal temperature, temperature of the steam after it has passed through SH II, and RH II metal temperature. The blue and red lines represent ten days’ worth of data in the healthy state, when the boiler is operating under normal conditions, and ten days’ worth of data recorded when the waterwall tube began to leak, respectively. It is clear that the generator’s active power and the corresponding thermocouple sensor data vary substantially during a waterwall tube leakage event as compared to the normal state of the boiler.

**Figure 7:**
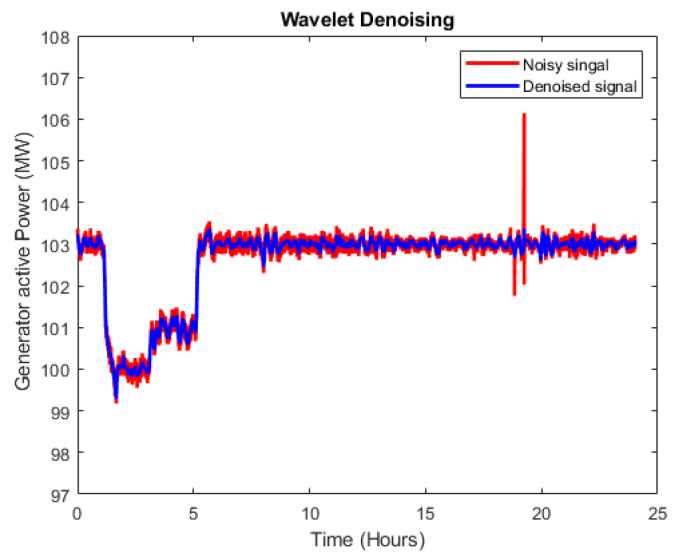
Denoising of the sensor data using the wavelet denoising method.

The wavelet analyzer toolbox in MATLAB [45] was used for wavelet denoising by choosing soft thresholding with five levels of decomposition. Figure 7 shows the efficacy of wavelet denoising using an example of the generator’s active power signal with a signal length of 24 h. The red line displays the noisy signal, whereas the blue line represents the denoised signal produced after removal of excess noise with the wavelet denoising technique.

### 4.2. Optimal Sensor Selection via Correlation Analysis

**Figure 8:**
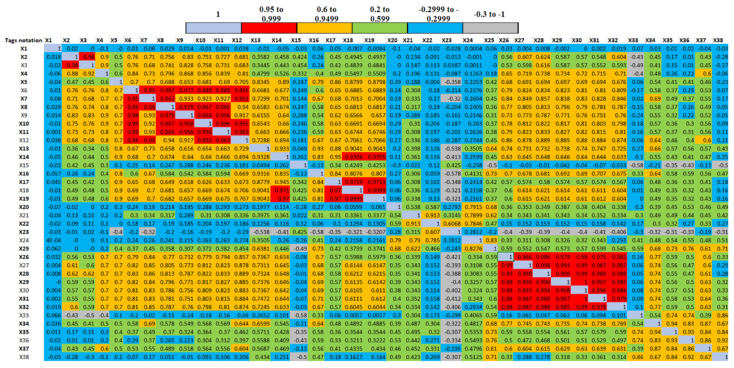
Correlation matrix exhibiting the correlation coefficient between all sensors’ data.

This section presents the results of correlation analysis. A Pearson correlation coefficient was computed for all sensor variables, and the resulting correlation matrix is shown in Figure 8. Two sensor variables are assumed to be highly correlated with each other when the value of the correlation coefficient is equal to or greater than 0.95, as represented in red in Figure 8.

**Figure 9:**
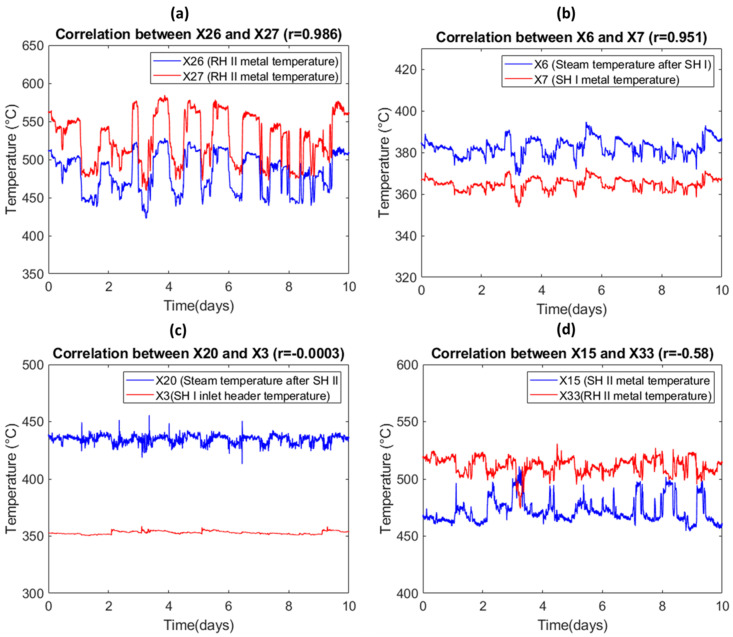
Correlations between different sensors with different correlation coefficients: (**a**) *r* = 0.951, (**b**) *r* = 0.606, (**c**) *r* = −0.003, (**d**) *r* = −0.58.

Figure 9 shows the data from several different sensors and exhibits the different correlation coefficient (*r*) values among the sensors. In Figure 9a,b, large correlations are observed between the sets of sensor signals, with correlation coefficients of 0.986 and 0.951. In Figure 9c, the *r* value of −0.0003 between X20 (steam temperature after SH II) and X3 (SH I inlet header temperature) indicates that there is no correlation between the data signals from these two sensors. In Figure 9d, X15 (SHII metal temperature) and X33 (RH II metal temperature) are negatively correlated with each other, with a correlation coefficient of −0.58.

Table 2 presents the highest correlation coefficients among the different pairs of data sensors. For example, X7, X8, X9, X10, X11, and X12 are highly correlated with X6, whereas X27, X28, X29, X30, X31, and X32 are highly correlated with X26. X6 represents the steam temperature after SHI, and it is highly correlated with the SH I metal temperature (sensors X7, X8, X9, X10, X11, and X12). X26 represents the RH I metal temperature, and it is highly correlated with the other RH I metal temperatures (X27, X28, X29, X30, and X31) and the RHI outlet steam temperature (X32). The classification may not be influenced by removing all but one of the highly correlated sensors. Therefore, these sensors are considered irrelevant and can be removed. Following the same procedure, 17 sensors are considered irrelevant, and 21 sensors are shortlisted and considered optimal sensors, as shown in Table 3.

### 4.3. Characteristics of the Dataset

The process variables are stored in the historical database of the distributed control system of the power plant, which has a sampling period of 1 sec. This study attempts to detect early and smaller tube leaks in a boiler waterwall tube; therefore, ten days’ worth of leak data (864,000 data points) from the early stage of the tube rupture are used for the machine learning analysis in addition to ten days’ worth of data from the healthy boiler (864,000 data points). Two cases (raw data and data from the optimal sensors) are employed in the machine learning model, and their performance is evaluated and compared. The raw data case consists of 38 input sensors, and the optimal data case after correlation analysis consists of 21 input sensors. Eighty percent of the individual sensor data are used as a training set, and the remaining twenty percent of the data are used as a test set. Table 4 provides information about the raw and optimal sensors datasets used for machine learning. 

### 4.4. Time Domain Statistical Feature Extraction

The leak-sensitive variables obtained from the SPP consist of time domain measurements. Direct analysis of these measurements will not provide satisfactory results. Therefore, to reduce the dimensions of the original data, evaluation of the statistical features of the time domain data was necessary. Statistical time domain features serve to not only decrease computational complexity but also separate signals with diverse structural integrity in the feature space. The time domain features used in this study consist of root mean square (RMS), variance (V), skewness (S), and kurtosis (K). Mathematical descriptions of the statistical time domain features are shown in Table 5.

### 4.5. Machine Learning Classifiers and Performance Evaluation

Four supervised machine learning classifiers (SVM, k-NN, NB, and LDA) are built and compared in terms of their performance in both the raw data case and the optimal sensors data case. Before a machine learning model is ready for application and implementation, its performance must be evaluated to validate its extrapolation ability and generalizability. There are several different validation techniques for performance evaluation, such as leave-one-out cross-validation, k-fold cross-validation, and bootstrapping [48]. In this study, 10-fold cross-validation is used to evaluate the training accuracy of the machine learning models.

The performance of the proposed machine learning-based integrated optimal sensor selection technique is compared to the raw data case in the machine learning model. Table 6 summarizes the results of SVM, k-NN, LDA, and naïve Bayes algorithms. Without implementing the sensor selection technique (raw dataset), SVM shows superior classification/testing accuracy (88.2%) compared to k-NN (85.5%), NB (84.2%), and LDA (86.8%). In the case of optimal sensor selection via correlation analysis, the performance increased slightly after removal of irrelevant and redundant features that caused overfitting of the machine learning models. The correlation analysis reveals an increase in classification performance (SVM: ↑2.3%, k-NN: ↑2.6%, NB: ↑1.5%, and LDA: ↑1.3%).

**Figure 10:**
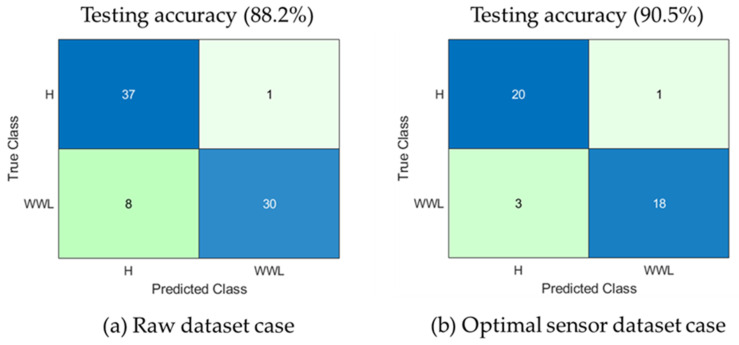
Confusion chart of the SVM-based machine learning model for the (**a**) raw dataset case and (**b**) optimal sensors dataset case.

Figure 10 shows the confusion matrix for the SVM classifier for both the raw data case and the optimal sensors case. H and WWL represent the healthy and waterwall tube leakage states of the boiler. The confusion chart shows the correct and incorrect predictions for both the raw and optimal data cases. The correct and incorrect predictions are on the diagonal and off-diagonal positions, respectively. SVM predicts the healthy data more accurately than the waterwall tube leakage data in both the raw and optimal sensors data cases.

High training accuracies accompanied by high testing accuracies show that the pre-trained classifiers would perform better in new instances of waterwall tube leakage. The results of the machine learning model show that the optimal sensor selection technique not only helped to identify the most sensitive sensor variables by reducing the number of sensors by 44% (from 38 to 21) but also increased the performance of the machine learning classifiers.

## 5. Conclusions

This study proposes a machine learning-based optimal sensor selection scheme to predict boiler waterwall tube leakage in an SPP. The multivariate algorithms used for data analysis are highly dependent on the number of input sensors. Selecting only the most informative sensors can save computational time and enhance model performance. Therefore, the optimal sensors were selected via correlation analysis of the leak-sensitive sensor data; this enabled use of only the most sensitive sensors to detect waterwall tube leakage and avoided data redundancy and use of irrelevant information due to the highly localized nature of the attached sensors. The correlation analysis was used to reduce the number of sensors by 44%, from 38 to 21 sensors. Furthermore, four supervised machine learning algorithms were developed, and their performance was evaluated and compared in both the raw data (38 sensors) and optimal sensors data (21) cases. The computational results indicate that the proposed SVM-based integrated optimal sensor selection process provided the highest accuracy among the models studied. This work suggests a simple and clear optimal sensor selection technique that is quick and easy to implement in SPPs.

## Figures and Tables

**Table 1:** Most sensitive sensor data from the Steam power plant used for boiler waterwall tube leakage detection.

ID	Description	Notation	ID	Description	Notation
P1CHA01GH001XQ01	Gen. Active Power	X1	P1HAH72CT003XQ01	Steam Temperature After SH II	X20
P1HAH55CT001XQ01	SH I Inlet Header Temperature	X2	P1HAH77CT001XQ01	SH III Metal Temperature	X21
P1HAH55CT002XQ01	SH I Inlet Header Temperature	X3	P1HAH77CT002XQ01	SH III Metal Temperature	X22
P1HAH55CT003XQ01	SH I Inlet Header Temperature	X4	P1HAH77CT003XQ01	SH III Metal Temperature	X23
P1HAH62CT001XQ01	Steam Temperature After SH I	X5	P1HAH77CT004XQ01	SH III Metal Temperature	X24
P1HAH62CT002XQ01	Steam Temperature After SHI	X6	P1HAH77CT005XQ01	SH III Metal Temperature	X25
P1HAH57CT001XQ01	SH I Metal Temperature	X7	P1HAJ15CT001XQ01	RH I Metal Temperature	X26
P1HAH57CT002XQ01	SH I Metal Temperature	X8	P1HAJ15CT002XQ01	RH I Metal Temperature	X27
P1HAH57CT003XQ01	SH I Metal Temperature	X9	P1HAJ15C003XQ01	RH I Metal Temperature	X28
P1HAH57CT004XQ01	SH I Metal Temperature	X10	P1HAJ15CT004XQ01	RH I Metal Temperature	X29
P1HAH57CT005XQ01	SH I Metal Temperature	X11	P1HAJ15CT005XQ01	RH I Metal Temperature	X30
P1HAH57CT006XQ01	SH I Metal Temperature	X12	P1HAJ15CT006XQ01	RH I Metal Temperature	X31
P1HAH67CT001XQ01	SH II Metal Temperature	X13	P1HAJ20CT001XQ01	RH I Outlet Steam Temperature	X32
P1HAH67CT002XQ01	SH II Metal Temperature	X14	P1HAJ35CT001XQ01	RH II Metal Temperature	X33
P1HAH67CT003XQ01	SH II Metal Temperature	X15	P1HAJ35CT002XQ01	RH II Metal Temperature	X34
P1HAH67CT004XQ01	SH II Metal Temperature	X16	P1HAJ35CT003XQ01	RH II Metal Temperature	X35
P1HAH67CT005XQ01	SH II Metal Temperature	X17	P1HAJ35CT004XQ01	RH II Metal Temperature	X36
P1HAH72CT001XQ01	Steam Temperature After SH II	X18	P1HAJ35CT005XQ01	RH II Metal Temperature	X37
P1HAH72CT002XQ01	Steam Temperature After SH II	X19	P1HAJ35CT006XQ01	RH II Metal Temperature	X38

**Table 2:** Correlation coefficients of highly correlated sensors.

Input Attributes	Highly Correlated Attributes	Correlation Coefficient (R)
X6 (Steam Temperature After SHI)	X7 (SHI Metal temperature)	0.951
X6	X8 (SHI Metal temperature)	0.987
X6	X9 (SHI Metal temperature)	0.977
X6	X10 (SHI Metal temperature)	0.989
X6	X11 (SHI Metal temperature)	0.989
X6	X12 (SHI Metal temperature)	0.965
X26 (RH I Metal Temperature)	X27 (RH I Metal Temperature)	0.986
X26	X28(RH I Metal Temperature)	0.986
X26	X29(RH I Metal Temperature)	0.979
X26	X30(RH I Metal Temperature)	0.982
X26	X31(RH I Metal Temperature)	0.975
X26	X32 (RH I Outlet Steam Temperature)	0.982

**Table 3:** List of optimal sensors determined by the correlation analysis.

#	Sensor ID	Sensor Description	Sensor Notation
1	P1CHA01GH001XQ01	Gen. active power	X1
2	P1HAH55CT002XQ01	SH I Inlet Header Temperature	X3
3	P1HAH55CT003XQ01	SH I Inlet Header Temperature	X4
4	P1HAH62CT001XQ01	Steam Temperature After SH I	X5
5	P1HAH67CT001XQ01	SH II Metal Temperature	X13
6	P1HAH67CT002XQ01	SH II Metal Temperature	X14
7	P1HAH67CT003XQ01	SH II Metal Temperature	X15
8	P1HAH67CT004XQ01	SH II Metal Temperature	X16
9	P1HAH72CT003XQ01	Steam Temperature After SH II	X20
10	P1HAH77CT001XQ01	SH III Metal Temperature	X21
11	P1HAH77CT002XQ01	SH III Metal Temperature	X22
12	P1HAH77CT003XQ01	SH III Metal Temperature	X23
13	P1HAH77CT004XQ01	SH III Metal Temperature	X24
14	P1HAH77CT005XQ01	SH III Metal Temperature	X25
15	P1HAJ15CT001XQ01	RH I Metal Temperature	X26
16	P1HAJ35CT001XQ01	RH II Metal Temperature	X33
17	P1HAJ35CT002XQ01	RH II Metal Temperature	X34
18	P1HAJ35CT003XQ01	RH II Metal Temperature	X35
19	P1HAJ35CT004XQ01	RH II Metal Temperature	X36
20	P1HAJ35CT005XQ01	RH II Metal Temperature	X37
21	P1HAJ35CT006XQ01	RH II Metal Temperature	X38

**Table 4:** Characteristics of the dataset used for machine learning.

Data Type	Input Sensors	No of Records	Train Set	Test Set	Target
Raw dataset	38	1,728,000	80%	20%	• Normal
Optimal dataset	21				• Leakage

**Table 5:** Statistical time domain features (*x* is the sensor signal).

Features	Mathematical Expression
Root mean square	RMS = ${\left(\frac{1}{N}\sum_{i=1}^{N}x_{i}^{2}\right)}^{\frac{1}{2}}$
Variance (V)	V = $\frac{\sum {\left(x_{i}-\bar{x}\right)}^{2}}{N-1}$
Skewness (S)	$S=\frac{1}{N}\overset{N}{\underset{i=1}{\sum }}{\left(\frac{x_{i}-\bar{x}}{\sigma }\right)}^{3}$
Kurtosis	$K=\frac{1}{N}\overset{N}{\underset{i=1}{\sum }}{\left(\frac{x_{i}-\bar{x}}{\sigma }\right)}^{4}$

**Table 6:** Performance evaluation and accuracy comparison.

Machine Learning Classification	Raw Data		Optimal Sensors Data	
Algorithms	Training Accuracy (%)	Testing Accuracy (%)	Training Accuracy (%)	Testing Accuracy (%)
SVM	90.8	88.2	92.9	90.5
k-NN	88.2	85.5	92.9	88.1
NB	86.8	84.2	88.1	85.7
LDA	89.5	86.8	90.5	88.1
